# Screening Fitness to Drive After Stroke Across Demographic Subgroups: A Systematic Review

**DOI:** 10.1177/15394492251344518

**Published:** 2025-06-16

**Authors:** April Vander Veen, Leaha Johnston, Jeffrey Holmes, Patricia Tucker, Liliana Alvarez

**Affiliations:** 1 Western University, London, Ontario, Canada

**Keywords:** occupational therapy, driving, stroke, systematic literature review

## Abstract

Return to driving is a valued activity among people who experience stroke. Health care providers, including occupational therapists, require evidence-based tools for driver screening post-stroke, validated for stroke with representation of diverse demographic subgroups. To identify tests supported in the literature predictive of fitness to drive after stroke and critically appraise the representativeness of extant research across demographic subgroups. A systematic literature review was conducted to address the objectives. Consistent with prior research, the Stroke Driver’s Screening Assessment and Trail Making Test-B were the most predictive of driver fitness. However, research has consistently underrepresented women, people younger than 55 years of age, and people from low-income countries. Further research is needed with (a) more detailed reporting of participant demographics and (b) increased representation of demographic subgroups within samples, to support culturally informed driver screening practices following stroke.

## Plain Language Summary

Screening Fitness to Drive Across Demographic Subgroups

Returning to driving is very important to people who have experienced a stroke. As a stroke can cause functional changes that make driving difficult, health care providers, including occupational therapists, must use tests to check fitness to drive. This review examined which tests are predictive of fitness to drive, for which subgroups of people who have had a stroke. Ideally, for a test to be appropriate to use with a patient, the test should be previously tested with study participants similar to the patient, for example, with similar age or education level. This review found that the Stroke Driver’s Screening Assessment and Trail Making Test-B are good predictors of fitness to drive after stroke. However, study samples in this research often did not include many women, people younger than 55 years of age, and people from low-income countries. More research is needed that includes people who are not represented in the studies to date.

## Introduction

Return to driving is an important priority among people who have experienced a stroke (Griffen et al., 2009; Patomella et al., 2009; White et al., 2012). Within a Canadian context, best practice guidelines stipulate all persons who have experienced a stroke who wish to return to driving should be screened for functional impairments using valid and reliable assessments to inform recommendations (Mountain et al., 2020). In the United States, most states have similar requirements prior to resumption of driving following a stroke (Winstein et al., 2016). Australia and New Zealand also have robust best practice guidelines for return to driving after stroke (2025). Similarly, many countries around the globe are developing or calling for the development of best practices and more research regarding resumption of driving after stroke, including within India (Gupta et al., 2024), Saudi Arabia (Almosallam et al., 2022), Chile (Riesco et al., 2018), and South Africa (Bryer et al., 2010). Occupational therapists are ideally positioned to evaluate fitness to drive and inform recommendations for return to driving after a stroke (World Federation of Occupational Therapists, 2019). However, therapists need access to appropriate screening tools to inform recommendations regarding fitness to drive post-stroke (Cammarata et al., 2017).

Previous systematic reviews have identified screening tools most predictive of fitness to drive among stroke populations (Devos et al., 2011; Hanna et al., 2017; Hird et al., 2014; Murie-Fernandez et al., 2014). However, the most recent review was published 7 years ago and focused on screening for visual-perceptual impairments only (Hanna et al., 2017). Furthermore, existing systematic reviews examined screening tools validated for stroke as a *diagnostic group* and the extent to which historically underrepresented *subgroups* are included in the extant literature is unknown. As return to driving is of high importance to patients, it is a priority to investigate which demographic subgroups are represented in the literature to adequately provide equity, diversity, and inclusion informed care. As such, the need for an updated systematic review that critically appraises the representation of different demographic subgroups of individuals who have experienced a stroke within the existing literature is warranted.

In stroke research more broadly, the Heart & Stroke Association (2023) released a report highlighting system-level changes required to address the gaps in stroke research and health care for specific demographic subgroups, particularly for women. Moreover, although the overall incidence of stroke is decreasing in high-income countries, stroke incidence is *increasing* for particular subgroups including women, young people, and people from low-income countries (Ekker et al., 2019; Leppert et al., 2022; Scott et al., 2022), the same subgroups of people often neglected in existing stroke research (Heart & Stroke Association, 2023). As screening tools are ideally used with the specific subgroup population for which they have been validated, it is reasonable to question the validity of currently recommended tools for subgroups who may not have been included in existing validation studies (Fujii, 2018).

Within the field of neuropsychology, where many tests for driver screening were developed, there are calls for demographically appropriate tests, normative data, and testing procedures to enhance the validity of assessment (Franzen et al., 2021; Fujii, 2018). The ECLECTIC framework was developed to inform culturally appropriate assessment practices. This framework considers (E) education and literacy, (C) culture and acculturation, (L) language, (E) economics, (C) communication, (T) testing situation, comfort and motivation, (I) intelligence conceptualization, and (C) context of immigration, as cultural moderators of test performance and provides strategies to enhance validity of results, and cultural sensitivity (Fujii, 2018). The ECLECTIC framework recommends referencing normative data as specific to the subgroup membership of the patient as possible and using functional tests when available, as functional assessments are less likely to misclassify a person’s real-life abilities (Fujii, 2018). Within occupational therapy, this attention to culture in assessment is echoed in recent practice frameworks that emphasize cultural safety, including interpreting findings within cultural contexts (Restall et al., 2022). To provide equity, diversity, and inclusion informed care, occupational therapists need access to research which report and include diverse demographic subgroups (Suarez-Balcazar et al., 2023).

Understanding subgroup representation is necessary for therapists and drivers to work together to identify the most appropriate tools that can adequately identify underlying abilities. Study participants for fitness to drive research are often sampled from people who present to driving assessment centers for formal driving assessments, (e.g., Chua et al., 2012; Korner-Bitensky et al., 2000; Selander et al., 2010). However, this only represents the group of people identified for further assessment, and such decisions are informed by earlier driver screening (Korner-Bitensky et al., 2010; Sangrar et al., 2018). Furthermore, formal driving assessments are often a costly out-of-pocket expense (Petzold et al., 2010). Thus, recruiting from assessment centers excludes people who were not offered, or cannot afford formal driving assessments.

As such, the purpose of the current research was twofold: (a) to update and extend previous reviews which identified office-based screening tools for predicting fitness to drive following stroke and (b) to critically appraise the representation of different demographic subgroups in extant fitness to drive research following stroke. Specifically, we assessed the representation of subgroups in which stroke incidence is *increasing* including women, people aged 55 and younger, people residing outside of high-income countries, and members of racialized communities (Ekker et al., 2019; Leppert et al., 2022; Scott et al., 2022). Racialized communities refer to persons who belong to visible, non-White minority groups including South Asian, Chinese, Black, Filipino, Arab, Latin American, Southeast Asian, West Asian, Korean, and Japanese (Statistics Canada, 2021).

## Method

### Design

The methodology for this systematic literature review followed Cooper et al. (2009). A study protocol was prepared and registered on PROSPERO (ID: (ID: CRD42023469594)), and results were reported per the Preferred Reporting Items for Systematic Review and Meta-Analysis (PRIMSA) guidelines (Page et al., 2021). The completed PRISMA checklist is presented in Supplemental Table A.

### Locating and Selecting Studies

To meet the research objectives and after consultation with the Western University Research Librarian, the following six databases were searched: Scopus, Medline (OVID), CINAHL, Embase, Cochrane, and PsychINFO. Searches were completed in October 2023 and updated in January 2025 to include any new publications through to December 2024. The search strategy combined keyword as well as subject heading searches utilizing Boolean operators for the following concepts: (a) drive, driving, driver; (b) fitness to drive, driver fitness; and (c) stroke (including all medical terms for stroke [e.g., cerebral vascular accident] and including all stroke subtypes [e.g., ischemic, hemorrhagic]). The detailed search strategy is presented in Table 1. Inclusion criteria for the review were studies that: employed a pass-fail outcome as the dependent variable for driver evaluation (on-road or via driving simulator as the validation method); specifically assessed people with stroke (or if the study included other diagnoses, outcomes for stroke population were reported separately); and the study population included drivers >18 years who held valid driver’s licenses prior to their stroke. All studies that met the inclusion criteria were included regardless of year of publication.

**Table 1.** Search Strategy.

Search label	Search terms
1	Automobile Driving/ or Automobile Driver Examination/ or Accidents, Traffic/
2	(fit* adj5 (drive* or “motor vehicle*” or operate or auto*)).tw.
3	1 or 2
4	Stroke Rehabilitation/ or Stroke/
5	stroke.tw
6	4 or 5
7	3 and 6

Studies were not excluded based on publication language. Attempts were made to obtain full-text translations for any studies published in languages not spoken fluently within the research team. Authors were contacted directly to inquire about translated copies. Following recommendations from Walpole (2019), when translations were not available, *Google Translate* was utilized to obtain translations and facilitate data extraction.

Google Scholar was also searched to increase the likelihood of identifying studies published in languages other than English. The “cited by” feature in Google Scholar was used to identify papers citing a seminal systematic review and meta-analysis (Devos et al., 2011). Devos et al. (2011) is cited 191 times by entries in Google Scholar and utilized similar inclusion criteria to the present study. Furthermore, the reference list of every included article was hand searched to identify relevant articles missed in database searches and Google Scholar.

Screening of papers was achieved via a three-step process. First, the Covidence platform was used to remove duplicates from the search output. Next, the references were uploaded to ASReview, an open-source machine learning platform (van de Schoot et al., 2021). ASReview uses active learning technology that adapts to researcher input to sort references according to relevance. As articles are manually identified as eligible or ineligible by the researchers, the list is continually re-sorted to bring most relevant titles to the top. Title and abstract screening was completed using ASReview by two independent researchers (AVV and LJ) for eligibility, and studies were included by consensus. A recent simulation study indicates that 100% of relevant studies are identified in the first 30% of articles sorted by ASReview (van de Schoot et al., 2021). Screening concluded after each independent researcher screened the first 30% of the titles, and when several consecutive titles (a further 10% of all titles found; *n* = 72) were marked irrelevant, thus screening a total of 40% of the titles. Disagreement involved a third researcher (LA) to determine eligibility. Finally, after title and abstract screening was completed in ASReview, the titles were uploaded to Covidence once again for a second round of title and abstract screening (as Covidence enables more specific tracking of reasons for exclusion). Studies were selected for full-text review and data extraction by consensus, any discrepancies were resolved by the third researcher (LA).

### Data Collection and Critical Appraisal

Data extraction was completed in Covidence by two independent researchers (AVV and LJ) and reviewed by the first author to consolidate the data extracted. Data extracted included: (a) screening tests utilized; (b) predictive validity statistics reported of the assessment/screening tool (including area under the curve, specificity, sensitivity, positive predictive value, negative predictive value, regressions and correlations, or between group differences between pass and fail on driving test) *as reported* (N.B. no calculations were completed for data extraction based on any raw data presented in the papers. For example, some studies included the 4x4 table, but only statistics explicitly reported by authors were extracted); (c) method for determining pass/fail (on-road or simulator); (d) recommended cut points for used screening tests; and (e) specific demographics of the study participants including: age, sex at birth, gender, geographical location of participants, racial/cultural identities and any other social demographics reported (i.e., income, education, employment status, where reported) and number of participants for each; and (f) geographic location of researchers (N.B. when demographic data does not clarify if reporting pertains to gender or sex at birth, it was presumed to be sex at birth as the driving literature tends to use both terms interchangeably). Quality appraisal followed a modified checklist of health care interventions originally developed by Downs and Black (1998). The modified checklist was developed by Duch et al. (2013) for studies that are not randomized trials. This checklist was further amended for the present study to exclude one item (regarding reporting precise probability values) which was not pertinent to all statistics reported in this review. The modified quality assessment checklist is presented in Supplemental Table 2. Quality appraisal was also completed by two independent researchers (AVV and LJ) and was reviewed by the first author to determine the final quality ratings.

### Analysis

To synthesize results, data extracted from included studies was presented in an evidence table (Table 2). To address objective one, to identify tests most predictive of fitness to drive after stroke, any screening tests with findings from more than one study was presented in another table (Table 3). This table was created to optimize utility for clinicians to review screening tests commonly used in the literature, research that supports and does not support its use, the study design and sample size of relevant studies, and demographics of the study participants across studies were combined to show the subgroups represented in examination of the test. Table 3 also includes an assigned grade of practice recommendations (Burns et al., 2011) for each test which provides practice recommendations for clinicians. The grade of practice recommendations is assigned based on the (a) level of evidence (e.g., prospective versus retrospective designs) and (b) the consistency of the findings across multiple studies (Supplemental Table 3). Grade A (Strong Recommendation) is assigned when there is level I evidence, or *consistent* findings across multiple level II, III, and IV studies. Grade B (Recommendation) is assigned with *generally consistent* level II, III, or IV evidence. Grade C (Option) is assigned for *inconsistent* level II, III, or IV evidence. Grade D (Option) is assigned with only level V evidence or little to no empirical evidence. Further descriptions of the implications for grade of practice recommendations are included in Table 3.

**Table 2.** Summary of Included Studies.

Author(s)	Country	Screening test(s)	Participant characteristics	Method, inclusion and exclusion criteria	Quality appraisal	Significant statistics reported
Akinwuntan et al. (2006)	Researchers: Belgium Participants: Belgium	Rey Osterreith Complex Figure; Stroke Driver’s Screening Assessment (SDSA); Useful Field of View (UFoV); visual acuity (monocular and binocular with Snellen eye chart), Kinetic Vision Test, Test for Attentional Performance (TAP)	Female = 11 Male = 57 Age: *M* = 53; *SD* = 13 Time since stroke: *M* = 15 months; *SD* = 18	Cohort Study Inclusion: 68 consecutive drivers who completed the pre-driver assessment	7/9	Statistical Analysis: Correlation, pass/fail on road test as dependent variable; Predictive model with pass/fail on road test as dependent variable Binocular Acuity *r_b_* = .2, *p* < .05. Divided Attention (Correct Response) *r* = .28, *p* <.05. TAP (Reaction Time) *r* = .26, *p* < .05; (Difference in error) *r* = .36, *p* < .01. Dot cancellation (SDSA) *r* = .22, *p* < .052. Square matrix direction (SDSA) *r* = .39, *p* < .01. Square matrix compass (SDSA) *r* = .32, *p* < .01. Road sign recognition (SDSA) *r* = .22, *p* <.05. SDSA (Discriminant Function) sensitivity = 79.4%; specificity = 94.1%, *PPV* = 93.1%
Akinwuntan et al. (2013)	Researchers: United States Participants: United States	Stoke Driver’s Screening Assessment (SDSA) Battery contains: Dot Cancellation (DC), Square Matrix Direction (SMD), Square Matrix Compass (SMC), Road Sign Recognition (RSR)	Stroke group: Female = 6 Male = 9 Control group: Female = 6 Male = 10 Age: Stroke group *M* = 52; *SD* = 12. Control group *M* = 40; *SD* = 16 Time since stroke: *M* = 5 months; *SD* = 2 Social identities: Education (stroke group) Highschool (*n* = 9), College (*n* = 5), Graduate school (*n* = 1)	Cohort Study Inclusion: first stroke <1 year, driver’s license, driving >16 km/week, MMSE >24, acuity 20/60, Barthel index > 60 Exclusion: neurological condition, visual field impairment, psychiatric disorder or seizures	6/9	Statistical Analysis: Predictive model with pass/fail on road test as dependent variable SDSA (Discriminant equation) Accuracy = 87% for stroke, 88% for control
Barco et al. (2014)	Researchers: United States; Australia Participants: United States	Motor-Free Visual Perceptual Test (MVPT); Trail Making Test A (TMT-A); Trail Making Test B (TMT-B); Snellgrove Maze Test; Useful Field of View (UFoV); Clock Drawing Test; visual acuity, visual fields, Peli-Robson contrast sensitivity chart, Short Blessed Test, Digit Span Forward and Backward test, Manual Muscle Testing, cervical ROM, grip strength (goniometer), Rapid Pace Walk, Nine Hole Peg Test, Braking Response Time	Female = 33 Male = 39 Age: *M* = 59.3; Range = 31-88 Social identities: Caucasian (*n* = 50; 70%), African American (*n* = 22; 30%). Years of education: *M* = 14.2, *SD* = 2.9	Cohort Study Inclusion: >10 years driving, having an informant, diagnosis stroke, English speaking, 0-13 on NIHSS Exclusion: active depression, unstable illness, severe physical limitations, sensory or communication impairments, sedating meds, had comprehensive driving evaluation in last 12 months, below vision standards for driver’s license	7/9	Statistical Analysis: Predictive model with pass/fail on road test as dependent variable Best model = TMT-A + Snellgrove Maze Test *ROC AUC* = .87. Based on cut point of .70 probability: true positive rate =70%; false positive rate = 70%; precision: 77%; accuracy = 74%; likelihood ratio = +6.0 (95% CI = [1.7-21.1]) Scores on Snellgove, TMT-A, TMT-B, Short Blessed Test, Clock Draw Test and UFoV were all able to distinguish between pass and fail on road test at *p*<.05 MVPT was not significant at *p* = .2
Björkdahl et al. (2015)	Researchers: Sweden Participants: Sweden	Stroke Driver’s Screening Assessment (Nordic SDSA) Battery contains: Dot Cancellation, Direction Task, Compass Directions, Recognition of Traffic Signs	Total = 110 Age and sex not reported	Cohort Study Inclusion: admitted to stroke unit or referred for screening Exclusion: no driver’s license, extensive personal care assistance, epilepsy, neglect, hemianopsia, needing an adapted vehicle	5/9	Statistical Analysis: Predictive validity of pass/fail on road test NorSDSA *PPV =* 78%; *NPV =* 84%
Bouillon et al. (2006)	Researchers: Canada Participants: Canada	Motor-Free Visual Perceptual Test (MVPT) Bells test, Cognitive Behavioural Driver’s Inventory (CBDI) which included: Bracy’s Computer Assisted Cognitive Rehabilitation subtests (visual reaction differential response, visual reaction differential response reversed, visual discrimination II, visual scanning III), Weschler Adult Intelligence Scale (picture completion and digit symbol subtests), Trail Making Test A&B, Brake Reaction Test, Visual Fields Score	Female = 37 Male = 135 Total = 172* * *n* =73 were stroke patients Age: *M* = 58.9; *SD* = 17.9 Social identities: Language, French (*n* = 140), English (*n* =2 4), Other (*n* = 8)	Historical Cohort Study Inclusion: those diagnosed with neurological condition who completed testing at their driving evaluation services between January 2000 and March 2003 Exclusion: medical conditions that legally precluded them from driving. Those who received their road test more than 75 days after the screening evaluation	7/9	Statistical Analysis: Predictive validity of pass/fail on road test CBDI: Left Cerebral Vascular Accident (CVA), using 45 as cut off: Sensitivity = 1; Specificity = 0.53; *PPV =* 0.57; *NPV =* 1. CBDI: Right CVA, using 45 as cut off: Sensitivity = 0.91; Specificity = 0.56; *PPV =* 0.7; *NPV =* 0.83 Recommended cut point = 45 on CBDI
Chua et al. (2012)	Researchers: Australia Participants: Australia	Road Law Test (RLT), Give Way Test (GWT), Visual Recognition Slide Test (VRST-USyd)	Female = 112 Male = 329 Age: *M* = 65.4 *SD* = 15.4; range = 56-78 Time since stroke: *M* = 12.3 months; *SD* = 20.9	Historical Cohort Inclusion: diagnosis of stroke, attended the Driver Assessment and Rehabilitation Centre between 1996 and 2010, had full driving assessment (on and off road) had a driver’s license prior to stroke Exclusion: people with driving related problems due to other conditions, such as macular degeneration	6/9	Statistical Analysis: Predictive model with pass/fail on road test as dependent variable; Correlations with pass/fail on road test as dependent variable RLT: *R*^2^ = 6.6% *p* <.01. GWT: *R*^2^ = 7.7%, *p* <.01. VRST-USyd: *R*^2^ = 7.6% *p* <.01 Most predictive model had time post-stroke + age + RLT + GWT + VRST-Usyd + gender (but gender and VRST did not independently produce significant contribution to predictive accuracy of the equation, *R*^2^ = 19.7%, *p* <.01. Correlations with road test result: RLT: *r_s_* = -0.26, *p* <.01. GWT: *r_s_* = -0.28, *p* <.01. VRST-USyd: *r_s_* = -0.28, *p* <.01
George et al. (2008)	Researchers: Australia Participants: Australia	Visual recognition slide test (VRST), Visual Scanning Analyser (VSA), Response time measures (RTM)	Female = 2 Male = 24 Age: *M =* 65.5; *SD* = 13.2 Time since stroke: *M* = 83.5 months	Cohort Study Inclusion: had stroke that compromised driving ability who wanted to return to driving, medical clearance to participate in a driving assessment, were recommended for a driving assessment	7/9	Statistical Analysis: Comparison of mean scores on tests between pass and fail groups on road test VRST: *F* =7.71 *p* = <.05. VSA field 5 (time): *F* = 4.41, *p* = <.05. RTM: Total processing time subtest, *F* = 3.80 *p* <.05. Inspection time for Two-Choice Incompatibility subtest, *F* = 4.74 *p* <.05)
George & Crotty (2010)	Researchers: Australia Participants: Australia	Stroke Driver’s Screening Assessment (SDSA; Battery included: Dot cancellation, Compass test, Road Sign Recognition Test); Useful Field of View (UFoV)* *UFoV generated cut points for overall risk were dichotomized into pass/fail. Pass = very low and low risk; fail = moderate, high to very high risk	Female = 14 Male = 52 Age: *M* = 65.9; *SD* = 8.4 Time since stroke: range = 10-2,190 days	Cohort Study Inclusion: diagnosis of stroke, previous driver, >18 years, able to provide written informed consent, adequate cognition to follow instructions and participate in assessment, were recommended to have an on-road assessment by the rehabilitation physician, had 120 degrees of vision in horizontal axis, medically stable, no complex car modifications	7/9	Statistical Analysis: Predictive validity of screening test with pass/fail on road test UFoV- Divided Attention subtest: Sensitivity = 85.7 (*CI* 59.85-100); Specificity = 69.4 *CI* (54.3-84.5), *PPV* = 35.3 *CI* (21-49.6); *NPV* = 96.2 *CI* (90.5-100); Accuracy = 77.5 UFoV; Selective Attention Subtest: Sensitivity = 42.9 *CI* (6.2-79.6); Specificity = 88.9 *CI* (78.7-99.1); *PPV =* 42.9 *CI* (28.2-57.6); *NPV* = 88.9 *CI* (79.5-98.3); Accuracy = 65.9 SDSA (original equation) Sensitivity = 71.4 (CI 37.9-100); Specificity = 77.8 (64.3-91.3); *PPV* = 38.5 (24-53); *NPV =* 93.3 (CI 85.5-100); Accuracy = 74.6
Holowaychuk et al. (2020)	Researchers: Canada Participants: Canada	Motor-Free Visual Perceptual Test (MVPT), Trail Making Test A (TMT-A), Trail Making Test B (TMT-B)	Female = 17 Male = 65 Age: *M* = 60.67; *SD* = 10.95; range = 25-84 Social identities: Language, English only (*n* = 50), bilingual (*n* = 8)	Historical Cohort Inclusion: primary neurological diagnosis, did both on and off-road assessments Exclusion: new drivers <5 years of experience	7/9	Statistical Analysis: Predictive model with pass/fail on road test as dependent variable TMT-B (time) predicted 76% of on-road drivers’ performance, -2log-likelihood = 59.6, exp b = 0.97. TMT-B variable (time) -0.36 (*p* = .013) only test that was significant in the model)
Kim et al. (2018)	Researchers: Korea Participants: Korea	Revised Cognitive Perceptual Assessment for Driving (CPAD2) Battery contains: Depth Perception (number correct), Depth perception (ms), Sustained Attention, Divided Attention, Stroop Test (number correct), Stroop Test (ms), Digit Span, Field dependency, Trail making Test-A (ms), Trail Making Test-B (ms)	Female = 5 Male = 25 Age: *M* = 50.77; *SD* = 10.77	Inclusion: Diagnosed with stroke > 3 months ago, who admitted to rehab and eligible for a license. Exclusion: Subjects who could not follow or understand instructions, not eligible for a driver’s license, epilepsy or seizures, apraxia or ataxia	6/9	Statistical Analysis: Predictive validity of screening test with pass/fail on road test, Correlations between screening tests and pass/fail on road test CPAD2, *PPV* = 90.9%, *NPV* = 50%. (N.B. pass [score of ≥ 53], borderline [score of >42 and <53], fail [ score of ≤42.]) Individual subtest correlations with road test outcome: Depth Perception Subtest, *r* = .555, *p* = .001. Sustained attention subtest, *r* = .482, *p* = .007. Divided attention, *r* = .67, *p* = .0001. Stroop Test (ms)*r* = .539, *p* = .002. Digit Span, *r* = .424, *p* = .02. Trail Making test-B (ms), *r* = .521, *p* = .0001
Klavora et al. (1995)	Researchers: Canada Participants: Canada	Dynavision	Female = 2 Male = 8 Age: Female *M* = 67, Male *M* = 62.1 range = only recruited 40-80 year olds Time since stroke: female: *M* = 11.5 months, male *M* = 9.75 months	Cohort Study Inclusion: stroke between 6 and 18 months ago, had visual and attention difficulties while driving, between 45 and 80 years of age and was deemed unsafe to drive in a driving assessment	6/9	Statistical Analysis: Comparison of mean scores on tests between “safe to resume driving” and “unsafe to resume driving” groups on road test Dynavision endurance task: *F* = 17.99, *p* <.001. Dynavision speed task: *F* = 16.65, *p* <.001. Dynavision choice visual reaction time: *F* = 6.02, *p* <.03
Klavora et al. (2000)	Researchers: Canada Participants: Toronto, Canada	Dynavision Performance Assessment Battery (DPAB). Subtests of battery included: Simple Dynavision task (SDT), Difficult Dynavision task (DDT), Complex Dynavision Task (CDT), Endurance Dynavision task (EDT). Pass Criterion: SDT pass ³50 responses per minute; DDT pass ³40 responses/min; CDT pass ³30 responses/sec; EDT pass ³195 correct responses/4min). Cognitive Behavioural Driver’s Inventory. Pass/fail criteria: <48 pass, 48-51 ambiguous, >52 unsafe	Female = 10 Male = 46 Age: *M* = 60.2; range = 44-82	Cohort Study Inclusion: >6 months post-stroke, had visual scanning or visual attentional problems, were recommended for an on-road driving assessment Exclusion: unstable medical condition, brain stem injury, history of psychiatric or substance abuse problems, poor vision, dementia, physical inability to execute motor sequences, current participation in a visual skills rehabilitation program, or did not have a driver’s license in the past	7/9	Statistical Analysis: Predictive model with pass/fail on road test as dependent variable Results for individual Subtests of DPAB: SDT: Accuracy = 66%; false positive rate = 4%; false negative rate = 30%. DDT: Accuracy = 68%; false positive rate = 4%; false negative rate = 28%. CDT: Accuracy = 68%; false positive = 4%; false negative rate = 28%. EDT: Accuracy = 75%; false positive rate = 7%; false negative rate = 18%. SDT and EDT: Accuracy = 77%; false positive = 7%; false negative rate =16%. Results for CBDI: CBDI, Accuracy = 66%; false positive (driver passes CBDI, but fails road test) = 4%, false negative (fails CBDI, but passes road test) = 30% CBDI and EDT: Accuracy = 100% *If a driver scores <47 CBDI and >195 on EDT they are 43.93 times more likely to pass to on-road test. *Authors recommend using EDT, which was the best individual predictor and a brief test
Kobayashi et al. (2017)	Researchers: Japan Participants: Japan	Trail Making Test A; Trail Making Test B; Mini-Mental State Examination (MMSE), Digit Span (DS), Tapping Span (TS), Visual Cancellation Task, (VCT) Auditory Detection Task (ADT), Symbol Digit Modalities Test (SDMT), Memory Updating Test (MUT), Paced Auditory Serial Addition Test (PASAT), Position Stroop Test (PST)	Female =28 Male = 153 Age: capable driver group *M* = 56 years, incapable group *M* = 62.8	Cohort Study Inclusion: regular driving for 10 years prior to stroke with driver’s valid license, >3 months since stroke, want to resume driving, no physical impairments impacting operating a vehicle Exclusion: unstable medical condition, history of epileptic seizure, dementia, mental illness, failure to meet local driver standards	4/9	Statistical Analysis: Predictive model with pass/fail on road test as dependent variable Symbol Digit Modalities Test (SDMT)*AUC =* 0.76, Sensitivity = 65%, Specificity = 79%. Logistic regression analysis: SDMT: Odds ratio = 1.054, *p* = .028. Scores on TMT-A, TMT-B, MMSE, DS, TS, VCT, ADT, SDMT, MUT, PASAT were able to distinguish between drivers who passed and failed on the road test at *p*<.01
Korner-Bitensky et al. (2000)	Researchers: United States and Canada Participants: United States and Canada	Motor-Free Visual Perceptual Test (MVPT)	Female = 54 Male = 215 Age: *M* = 63.6; *SD* = 12.5 Time since stroke: *M* = 6.9 months; *SD* =11	Historical Cohort Inclusion: diagnosed with stroke and referred for driver testing Exclusion: no hemianopsia or uncorrected visual impairment, class IV cardiac status, uncontrolled seizures	6/9	Statistical Analysis: Predictive validity of pass/fail on road test MVPT: *PPV =* 60.9%; *NPV =* 64.2%
Lundberg et al. (2003)	Researchers: Sweden, Norway and Finland Participants: Norway and Sweden	Nordic Stoke Driver’s Screening Assessment (NorSDSA) Battery included: Dot cancellation test, Directions test, Compass test, Road Sign Recognition Test	Female = 10 Male = 87 Age: *M* = 63; *SD* = 12.45; range = 26-85 Time since stroke: *M* = 1.1 years; *SD* = 1.45 years; range = 0.1-8 years Social identities: Education, primary (*n* = 33, 40%), secondary (*n* = 30; 37%), university (*n* =19, 23%)	Cohort Study Inclusion: diagnosed with stroke and at one of three hospital sites Exclusion: conditions that legally preclude driving, epilepsy, homonymous hemianopsia, one site only recruited people between 25-65 years of age, 3-9 months form first stroke, no excessive alcohol consumption	5/9	Statistical Analysis: Predictive validity of a discriminant function for pass/fail on road test NorSDSA- original discriminant function (Nouri & Lincoln, 1992), Sensitivity = 70%; Specificity = 67%; Accuracy = 68% NorSDSA new model from current study with random sample (*n* = 49), Sensitivity: 74%; Specificity: 77%; Accuracy: 75.5% NorSDSA new model from current study with entire sample (*n* = 97), Sensitivity = 36%; Specificity = 100%; Accuracy = 81%
Lundqvist et al. (2000)	Researchers: Sweden Participants: Sweden	Neuropsychological test battery: Pen-and-paper (Trail Making Test A; Trail Making Test B; Digit Symbol; Color Word Test [CWT]) Auditory tests (Paced Auditory Serial Addition Test [PASAT], Listening Span Test) Computerized tests: (Finger tapping, K test, Reaction Time tests, simultaneous capacity test, Wisconsin Card Sorting Test) Driving Simulator Tests: Speed, Lateral Position, Complex Reaction Time, Time to Collision, Distance to Collision	Female = 9 Male = 21 Age: *M* = 68.3; *SD* = 4.8, range = 60-75 years Time since stroke: *M* = 8.6 months; range = 3-14 months Social identities reported: education *M* = 9.1 years; *SD* = 3.5	Cohort Study Inclusion: CVA admitted to hospital who can perform the included tests and on-road driving evaluation, and those who had complete data sets Exclusion: epilepsy, hemianopsia, aphasia, apraxia, physical disabilities, no license, did not drive before, incomplete data, people unable to complete the tests due to impairments/limitations (stress, dysphasia)	6/9	Statistical Analysis: Predictive model with pass/fail on road test as dependent variable Among all the tests in the predictive model, ComplexReaction Time (driving simulator test) was a significant predictor (c^2^ = 15.76, *p* < .01) and this model correctly classified 83% of participants Stepwise logistic regression of driving simulator tests revealed Complex Reaction Time for Visual Stimuli and Distance to Collision were significant variables (c^2^ = 14.99, *p <* .01). This model overall classified 85% of subjects correctly Among tests in neuropsychological test battery, Cognitive Processing (Color Word Test, Listening Span test, PASAT and K Test) discriminated significantly between pass and fail groups, *t*(56) = 3.08; *p* < .01
Mazer et al. (1998)	Researcher Canada Participants: Canada	Motor-Free Visual Perceptual Test (MVPT) Trail Making Test A (TMT-A) Trail Making Test B (TMT-B); Complex Reaction Time (CRT), Single Letter Cancellation Test (SLCT), Double letter cancellation test (DLCT), The Money Road map Test of direction sense, Bells Test, Charron Test	Female = 21 Male = 63 Age: *M* = 60.8 *SD* =11.9; range = 27-84 Time since stroke: *M* = 10.4 months; *SD* = 15.8; range = 1-9.6 months	Cohort Study Inclusion: Stroke patients admitted to hospital, Exclusion: Homonymous hemianopsia, primary visual impairment that cannot be corrected by glasses, class IV cardiac status, uncontrolled seizures	7/9	Statistical Analysis: Predictive validity of screening tests for pass/fail on road test; Predictive model with pass/fail on road test as dependent variable MVPT: *PPV =* 86.1%; *NPV =*58.3%. TMT-A: *PPV =* 80%; *NPV =* 41.90%. TMT-B: *PPV =* 85.2%; *NPV =* 48.10%. SLCT (<5 errors): *PPV =* 78.90%; *NPV =* 44.60%. Bells Test (<4 errors): *PPV =* 77.80%; *NPV =* 43.90%. CRT (<118 points) *PPV =* 74.40%; *NPV =* 56.10%. Road Map (<4 errors) *PPV =* 72.10%; *NPV =* 52.60%. DLCT (<5 errors): *PPV =* 64.90%; *NPV =* 42.60%. Charron (<5 errors) *PPV =* 72.40%; *NPV = 5.5%.* Univariate logistic regression: CRT (<118, ³118s): EC = 1.11, *OR* = 3.03. MVPT (<30, ³30) EC = 2.16, *OR* = 8.68. SLCT: (< 5, ³ 5 errors) *EC* = 1.11, *OR* = 3.02. DLCT: (< 5, ³5 errors) *EC* = .31; *OR* = 1.37. Road map (<4, ³ 4 errors) *EC* = 1.00, *OR* = 2.71. TMT-A (<1 error, ³1 error) *EC* = 1.06, *OR* = 2.88, TMT-B (<3, ³ 3 errors) *EC* = 1.78, *OR* = 5.96. Bells Test (<4, ³ 4 errors) *EC* =1.01, *OR* = 2.74. Charron (<5, ³5 errors) *EC* = .78, *OR* = 2.19
Munin et al. (2023)	Researchers: Malaysia Participants: Malaysia	Malaysian Stoke Driver’s Screening Assessment (MySDSA) Battery included: Dot Cancellation, Square Matrix Direction, Square Matrix Compass, Road Sign Recognition	Female = 5 Male = 15 Age: *M* = 50; *SD* = 10.17 Social identities: Malaysian (*n* = 9; 45%), Indian (*n* = 2; 10%), Chinese (*n* = 9; 45%). Education: primary (*n* = 1, 5%), secondary (*n* = 8; 40%), tertiary (*n* = 11; 55%). Employed (*n* = 6; 30%), unemployed (*n* =14; 70%)	Cohort Study Inclusion: diagnosis of stroke, driver’s license, were driving > 3 times/week, >24 MMSE, >18 MoCA, >75 Barthel index Exclusion: neurological disease(s), <18 or >70 years, psychiatric disorder, conditions that preclude driving	5/9	Statistical Analysis: Predictive validity of screening tests for pass/fail on road test MySDSA (positive test = subject unfit to drive) Sensitivity = 62.5; Specificity = 75; *PPV =* 15.6; *NPV =* 96.4; Accuracy = 74%; *AUC =* 0.688, *p* = .095
Nouri et al. (1987)	Researchers: United Kingdom Participants: Sweden	Rey Osterreith Complex Figure; Cube Copy; Road Sign Recognition; Dot cancellation, Four Choice Reaction Time, What’s in the Square, What else is in the Square, Pursuit Rotor, Token Test, Titmus Vision Tester and Perimeter, Hand Sequencing Task, Recognition Memory Test-Faces, Hazard Recognition Task	Female = 3 Male = 36 Age: *M* = 54; *SD* = 10; range = 33-75 Time since stroke: range = 1.4-14 months	Cohort Study Inclusion: stroke, 6 weeks post-stroke, full licenses, driving in the 3 months before stroke Exclusion: medical or psychiatric condition precluding driving, no visual exclusion except blindness	5/9	Statistical Analysis: Predictive validity of a discriminant function for pass/fail on road test Discriminant function analysis included: Dot Cancellation, Rey Osterreith Complex Figure, What else is in the square, Pursuit Rotor, Vision Tester and Perimeter, Token Test, Recognition Memory Test, Cube Copy, Hazard Recognition Task, Accuracy = 94%
Nouri & Tinson (1988)	Researchers: United Kingdom Participants: United Kingdom	Simulator assessment	Female = 2 Male = 36 Age: *M* = 59; *SD* = 10; range = 33-75 years	Cohort Study Inclusion: at least 8 weeks from stroke, full driver’s license Exclusion: medical or psychiatric condition precluding driving, epilepsy	5/9	Statistical Analysis: agreement between impression from driving simulator (pass/borderline/fail), with ratings on on-road test (pass/borderline/fail) Kappa coefficients (K) calculated to examine the agreement with ratings from driving simulator and on-road test revealed poor level of agreement (*K* = 0.29). (K <.40 = poor agreement)
Nouri & Lincoln (1992)	Researchers: United Kingdom Participants: United Kingdom	Token test, visual acuity, recognition memory test, faces, what’s in the square, what else is in the square, cube copy, dot cancellation, Rey Osterreith Complex Figure and recall, pursuit rotor, Titmus vision tester and perimeter, road sign recognition test, hazard recognition task,	Female = 4 Male = 36 Age: *M* = 61.1; *SD* = 14.1; range = 37-79 Time since stroke: *M* = 33.1 months; *SD* = 40.7 months; range = 5-225 months	Cohort Study Inclusion: current full driver’s license, Exclusion: medical or psychiatric condition that prevented driving, hemianopsia	5/9	Statistical Analysis: Predictive validity of a predictive model for pass/fail; Comparison of mean scores between “pass”, “fail” and “borderline” groups on road test Discriminant model: Accuracy = 82.2% in *n* = 45 subset of sample, 79.4% for the remaining *n* = 34 Significant group differences for: Face recognition *F* = 3.53. *p* <.05. Dot cancellation (misses) *F* = 6.34, *p* <.05. Rey Osterreith Complex Figure: *F* = 5.23, *p*<.05. Pursuit rotor 15 rpm *F* = 6.22, *p*<.05. Token test *F* = 4.75, *p* < .05. Cube copy *F* = 15.97, *p* <.00. Dot cancellation time *F* = 9.45, *p* <.001. Rey Osterreith Complex Figure recall *F* = 8.92, *p* <.001. “What else is in the square?” *F* = 24.35, *p* <.001. Pursuit rotor 10 rpm *F* = 9.27, *p* .001. Road sign recognition *F* = 16.84, *p* <.001. Hazard recognition *F* = 13.40, *p* <.001
Nouri & Lincoln (1993)	Researchers: United Kingdom Participants: United Kingdom	Stroke Driver’s Screening Assessment (SDSA) Battery included: Dot Cancellation (DC), Square Matrix Direction (SMD), Square Matrix Compass (SMC), Road Sign Recognition (RSR)	SDSA group: Female = 4 Male = 23 Control group: female = 2 male = 23 Age: SDSA group: *M* = 60.2 Control group: *M* = 58.8 Time since stroke: SDSA: *M* = 23 weeks. Control, *M* = 44.4 weeks	Cohort Inclusion: diagnosis of stroke, had been driving in the 3 months prior to stroke, full driver’s license Exclusion: conditions that would legally preclude driving, e.g. epilepsy	3/9	Statistical Analysis: Predictive validity of a discriminant function for pass/fail on road test SDSA: Sensitivity = 89% (to detect passes); Specificity = 75% (to detect fails); *PPV =* 67 (of predicting a pass); *NPV =* 89 (of predicting a fail); Accuracy = 81%; Likelihood ratio = 6.0 (CI 1.5-24).
Selander et al. (2010)	Researchers: Sweden Participants: Sweden	Nordic Stoke Driver’s Screening Assessment (NorSDSA) Battery included: Dot Cancellation, Directions, Compass, Road Sign Recognition	Female = 8 Male = 68 Age: *M* = 65.3; *SD* = 9.8; range = 43-85 Time since stroke: > 6 months Social identities: education *M* = 10.6 years; *SD* = 3.4; range = 6-25 years	Historical Cohort Inclusion: diagnosis of stroke who were referred for a driving assessment	7/9	Statistical Analysis: Predictive validity of predictive model for pass/fail on road test NorSDSA (with cut off of zero): Sensitivity = 42%; Specificity = 76%; Accuracy = 62%. Discriminant analysis including dot cancellation (time and errors), compass, road sign recognition (3 and 5 min) Accuracy: 62%
Söderström et al. (2006)	Researchers: Sweden Participants: Sweden	Trail Making Test B, Reaction Time Test, Finger Tapping Test, Wisconsin Card Sorting Test, Rey Osterreith Complex Figure, Digit Symbol Test, Traffic Theory Knowledge Test	Female = 2 Male = 32 Age: *M* = 54; *SD* = 8.8; range = 28-67 Social identities: Education: secondary (*n* = 19; 55.69%), post-secondary (*n* = 15; 44.1%)	Cohort Study Inclusion: 25-67 years, first stroke, valid license, drove >2,000 km/year Exclusion: history of drug or alcohol abuse, psychiatric or medical condition, epilepsy, hemianopsia, serious aphasia	6/9	Statistical Analysis: Predictive model with pass/fail on road test as dependent variable Among all predictors in the model (entry set at *p* <.05), only age and driving experience were significant. Driving experience was the biggest predictor of variance (30.2%) compared to age (18.1%).
Sommer et al. (2010)	Researchers: Austria, Germany Participants: Austria, Germany	Test battery from XPSV (Adaptive Matrices Test, Determination Test, Reaction Test, Tachistoscopic Traffic Perception Test (TTPT), Cognitrone, Peripheral Perception test, Vienna Risk-Taking Test Traffic (VRTT), Inventory of Driving-Related Personality Traits	Female = 21 Male = 88 Age: *M* = 51.39; *SD* = 8.92; range = 24-68 Time since stroke: *M* = 12.39 months; *SD* = 20.24; range = 1-120 Social identities: “Education level” 2 (*n* = 11), 3 (*n* = 62), 4 (*n* = 11), 5 (*n* = 24)	Cohort Study Inclusion: diagnosis of stroke, driver fitness was questioned, driving >5 years Exclusion: visual impairments, heart disease, seizures, syncope, mental disorders, kidney disease, substance abuse, medications that impair driving	7/9	Statistical Analysis: Predictive model with pass/fail on road test as dependent variable Logistic regression model was significant (2 log-likelihood = 42.995; c^2^ [13] = 71.922, *p* < .001, Nagelkerke *R*^2^ = .741). Sensitivity = 83.3%; Specificity = 97.6%; Accuracy = 94.5%. Predictors in model *p* = < .05: Determination test (complex choice reaction subtest), *p* = .002. TTPT (perceptual speed) *p* = .037. VRTT: (subjective accepted level of risk), *p* = .001. Inventory of Driving-Related Personality Traits (social responsibility), *p* = .037.
Sotokawa et al. (2024)	Researchers: Japan Participants: Japan	Brunnstrom Recovery Stage (BRS), Functional Independence Measure (FIM), Letter Cancellation Task (LCT), Star Cancellation task (SCT), Symbol Digit Modality Task (SDMT)	Non-return to driving group: Female = 3 Male = 10 Age: not reported Return to driving group: Female = 2 Male = 14 Age = not reported	Cohort Study Inclusion: right hemispheric stroke >1 month, independent function, > 18 years of age, sufficient communication. Exclusion: epilepsy, neurological disorders, visual impairment, <1 year driving experience	7/9	Statistical Analysis: Comparison of mean scores on tests between pass and fail groups on road test; predictive validity of cut points for pass/fail LCT: cut point = 39.5 (Sensitivity = 0.62 Specificity = 0.50; *AUC* = 0.57, 95% CI (0.32-0.78) SCT: cut point = 53.5 (Sensitivity = 0.62; Specificity = 0.250; *AUC* = 0.409, 95% CI (0.23-0.59)
Unsworth et al. (2019)	Researchers: Australia Participants: Australia	OT-DORA Battery (Visual Acuity chart [Snellen], visual confrontation test, Motor sequences Screen-selected, Test of Proprioception- Lower limb, Berg Balance Scale, Motricity Index, Simulated Accelerator Brake Test, Right Heel pivot test, Road Law Road Craft Test, Occupational Therapy-Drive Home maze test, Mini-Mental State Examination)	Female = 48 Male = 100 Age: *M* = 65.36; *SD* = 14.72; range = 20-95	Cohort Study Inclusion: diagnosis of stroke, meeting vision standards for Australia,	7/9	Statistical Analysis: Predictive model with pass/fail on road test as dependent variable OT-DORA: *AUC* = 0.831; Specificity = 87.5%; Sensitivity = 72.2%. Participants tend to fail the on-road test if Mini-Mental State Examination is <22.557. Similarly, on “road law road craft” test, if participants score <20.5 typically fail on-road test, and > usually pass

*Note. M =* mean; *SD =* standard deviation; PPV = positive predictive value; NPV = negative predictive value; r_b_ = biserial correlation coefficient; *r* = Pearson correlation coefficient; MMSE = Mini-Mental State Examination; ROC = receiver operator characteristic; AUC = area under the curve; *SE* = standard error; OR = odds ratio; CI = confidence interval; EC = estimated coefficient.

**Table 3.** Driver Screening Tests, Studies That Support and Do Not Support the Use of the Tests, Individual Study Details (Design and Sample Size), and Combined Participant Demographics.

Screening test	Research NOT supporting use of the test: Relevant studies, study details, and combined participant demographics	Research supporting use of the test: Relevant studies, study details, and combined participant demographics	Grade of practice recommendation
Cognitive Behavioural Driver’s Inventory (CBDI)		1. Bouillon et al. (2006) Historical (*n* = 172) 2. Klavora et al. (2000) Cohort (*n* = 56)	Grade C: Option *Clinicians should be flexible in their decision-making* *regarding appropriate practice, although they may set bounds* *on alternatives; patient preference should have a substantial influencing role*
		Number of Participants: Total = 228 Female = 47 Male = 181	
		Age: *M* = 59.5 years *SD =* 0.6; Range = 44-82 (Klavora reported)	
		Country of participants: Canada	
		Social Identities reported: Bouillon et al. (2006): French: (*n* = 140); English (*n* = 24); Other (*n* = 8)	
Driving simulator test: Complex reaction time		1. Lundqvist et al. (2000) Cohort (*n* = 30) 2. Mazer et al. (2003) Historical (*n* = 84)	Grade C: Option *Clinicians should be flexible in their decision-making* *regarding appropriate practice, although they may set bounds* *on alternatives; patient preference should have a substantial* *influencing role*
		Number of Participants: Total = 114 Female = 30 Male = 84	
		Age: Weighted Mean = 62.8 years; *SD* = 3.3; Range = 27-84	
		Country of Participants: Sweden, Canada	
		Social Identities reported: Lundberg et al. (2003): *M* = 9.1 years of education; *SD* =3.5	
Dynavision Performance Assessment Battery (DPAB)		1. Klavora et al. (2000) Cohort (*n* = 56) 2. Klavora et al. (1995) Cohort (*n* = 10)	Grade D: Option *Clinicians should consider all options in their decision making* *and be alert to new published evidence that clarifies the* *balance of benefit versus harm; patient preference should have a substantial influencing role*
		Number of Participants: Total = 66 Female = 12 Male = 54	
		Age: Weighted Mean = 60.5; *SD* = 0.7; Range = 44-82* (*Klavora et al. [1995] did not report, recruited 40-80 year olds)	
		Country of participants: Canada	
		Social Identities reported: None	
Rey Osterreith Complex Figure	1. Söderström et al. (2006) Cohort (*n* = 34)	1. Akinwuntan et al. (2006) Cohort (*n* = 68) 2. Nouri et al. (1987) Cohort (*n* = 39) 3. Nouri & Lincoln (1992) Cohort (*n* = 40)	Grade C: Option *Clinicians should be flexible in their decision-making* *regarding appropriate practice, although they may set bounds* *on alternatives; patient preference should have a substantial* *influencing role*
	Female = 2 Male = 32 Age: *M* = 54; *SD* = 8.8; Range = 28-67	Number of Participants: Total = 147 Female = 14 Male = 133 Age: Weighted Mean = 55.5 years; *SD* = 3.5; Range = 33-79* (*Akinwuntan et al. [2006] did not report)	
	Country of participants: Sweden	Country of Participants: Sweden, Belgium	
	Social Identities reported: secondary education (*n* = 19); post-secondary (*n* = 15)	Social Identities reported: None	
Motor-Free Visual Perceptual Test (MVPT)	1. Barco et al. (2014); Cohort (*n* = 72) 2. Bouillon et al. (2006); Historical cohort (*n* = 172) 3. Holowaychuk et al. (2020); Historical cohort (*n =* 82) 60.67	1. Korner-Bitensky et al. (2000); Historical Cohort (*n* = 269) 2. Mazer et al. (1998); Historical Cohort (*n* = 84)	Grade C: Option *Clinicians should be flexible in their decision-making* *regarding appropriate practice, although they may set bounds* *on alternatives; patient preference should have a substantial* *influencing role*
	Number of participants: Total = 326 Female = 87 Male = 239	Number of participants: Total = 353 Female = 75 Male = 278	
	Age: Weighted Mean = 59.43 years; *SD =* 0.87; range = 25-88* (*Bouillon did not report range)	Age: Weighted *M* = 62.93 years *SD* = 1.94, range = 27-84* years (*Korner-Bitensky did not report range)	
	Country of Participants: Canada	Country of Participants: Canada, United States	
	Social Identities Reported: Bouillon et al. (2006): French (*n* = 140); English (*n* = 24); Other (*n* = 8) Holowaychuk et al. (2020): English (*n* = 50); bilingual (*n* = 8)	Social Identities Reported: Barco et al. (2014): Caucasian (*n* = 50), African American (*n* = 22), *M* = 14.2 years of education, *SD* = 2.9	
Nordic Stroke Driver’s Screening Assessment (NorSDSA)		1. Björkdahl et al. (2015) Cohort (*n* = 110) 2. Lundberg et al. (2003) Cohort (*n* = 97) 3. Selander et al. (2010) Historical (*n* = 76)	Grade A: Strong Recommendation *Clinicians should follow a strong recommendation unless a clear and compelling rationale for an alternative approach is present*
		Number of participants: Total = 283 Female = 18* (*Björkdahl did not report) Male = 155	
		Age: Weighted Mean = 64* years; *SD* = 1.4 (*Björkdahl did not report); Range = 26-85	
		Country of participants: Norway and Sweden	
		Social Identities Reported: Lundberg et al. (2003): primary education (*n* = 38); secondary education (*n* = 29), university (*n* = 18) Selander et al. (2010): *M* = 10.6 years of education; *SD* = 3.4; Range = 6-25 years of education	
Stroke Driver’s Screening Assessment (SDSA)		1. Akinwuntan et al. (2006) Cohort (*n* = 68) 2. Akinwuntan et al. (2013) Cohort (*n* = 31) 3. George & Crotty (2010) Cohort (*n* = 66) 4. Munin et al. (2023) Cohort (*n* = 20): Malaysian version 5. Nouri & Lincoln (1993) Cohort (*n* = 52)	Grade A: Strong Recommendation *Clinicians should follow a strong recommendation unless a clear and compelling rationale for an alternative approach is present*
		Number of participants: Total = 237 Female = 48 Male = 189	
		Age: Weighted *M* = 56.8 years; *SD* = 7.3; Range not reported	
		Country of Participants: United Kingdom, Australia, United States, Malaysia and Belgium	
		Social Identities Reported: Akinwuntan et al. (2013): high school (*n* = 9), college (*n* = 5), graduate (*n* = 1) (in stroke group) Munin et al. (2023): Malaysian (*n* = 9); Indian (*n* = 2); Chinese (*n* = 9). Primary education (*n* = 1); secondary (*n* = 8); tertiary (*n* = 11). Employed (*n* = 6); unemployed (*n* = 14)	
Trail Making Test-A	1. Holowaychuk et al. (2020) Historical (*n* = 82) 2. Lundqvist et al. (2000) Cohort (*n* = 30)	1. Barco et al. (2014) Cohort (*n* = 72) 2. Kobayashi et al. (2017) Cohort (*n* = 181) 3. Mazer et al. (1998) Historical (*n* = 84)	Grade C: Option *Clinicians should be flexible in their decision-making* *regarding appropriate practice, although they may set bounds* *on alternatives; patient preference should have a substantial* *influencing role*
	Number of participants: Total = 112 Female = 26 Male = 86 Age: Weighted Average = 62.7; *SD* = 3.4; Range = 25-84	Number of participants: Total = 337 Female = 82 Male = 255 Age: Weighted Average = 59; *SD* = 2.6; Range: 27-88 (only reported in Mazer)	
	Country of participants: Sweden, Japan, and Canada	Country of participants: United States, Canada, Japan	
	Social Identities Reported: Lundqvist et al. (2000): *M* = 9.1 years of education; *SD* = 3.5 Holowaychuk et al. (2020): English only (*n* = 50), bilingual (*n* = 8)	Social Identities Reported: Barco et al. (2014): Caucasian (*n* = 50), African American (*n*= 22), *M* = 14.2 years of education, *SD* = 2.9	
Trail Making Test- B	1. Lundqvist et al. (2000) Cohort (*n* = 30) 2. Söderström et al. (2006) Cohort (*n* = 34)	1. Barco et al. (2014) Cohort (*n* = 72) 33+39 59.3 2. Holowaychuk et al. (2020) Historical (*n* = 182) 3. Kim et al. (2018) Cohort (*n* = 30) 4. Kobayashi et al. (2017) Cohort (*n* = 181) 5. Mazer et al. (1998) Historical (*n* = 84)	Grade B: Recommendation *Generally, clinicians should follow a recommendation but* *should remain alert to new information and sensitive to patient* *preferences*
	Number of participants: Total = 64 Female = 11 Male = 53 Age: Weighted average = 60.7 years; *SD* = 7.2	Number of participants: Total = 549 Female = 80 Male = 345 Age: Weighted average = 59.1 years *SD* = 2.94; Range = 27-88 (only reported for Mazer and Barco)	
	Country of participants: Korea, Sweden, Japan, Canada, and United States	Country of participants: United States, Canada, Korea, Japan	
	Social Identities Reported: Lundqvist et al. (2002): *M* = 9.1 years of education; *SD* = 3.5 Söderström et al. (2006): Secondary education (*n* = 19), post-secondary (*n* = 15)	Social Identities Reported: Holowaychuk: English (*n* = 50), Bilingual (*n* = 8) Barco et al. (2014): Caucasian (*n* = 50), African American (*n* = 22), *M* = 14.2 years of education; *SD* = 2.9	
Useful Field of View (UFoV)	1. Akinwuntan et al. (2006) Cohort (*n* = 68)	1. Barco et al. (2014) Cohort (*n* = 72) 2. George & Crotty (2010) Cohort (*n* = 66)	Grade C: Option *Clinicians should be flexible in their decision-making* *regarding appropriate practice, although they may set bounds* *on alternatives; patient preference should have a substantial* *influencing role*
	Female = 11 Male = 57 Age: *M* = 53; *SD* = 13; Range not reported	Number of Participants: Total = 138 Female = 47 Male = 91 Age: Weighted *M* = 62.46* years; *SD =* 3.3; Range 31-88 (*only Barco et al. [2014] reported)	
	Country of participants: Belgium	Country of Participants: Australia and United States	
		Social identities reported: Barco et al. (2014): Caucasian (*n* = 50), African American (*n* = 22), *M* = 14.2 years of education, *SD* = 2.9	
Visual recognition slide test (VRST)		1. Chua et al. (2012) Historical (*n* = 441) 2. George et al. (2008) Cohort (*n* = 26)	Grade C: Option *Clinicians should be flexible in their decision-making* *regarding appropriate practice, although they may set bounds* *on alternatives; patient preference should have a substantial influencing role*
		Number of Participants: Total = 467 Female = 114 Male = 353 Age: Weighted *M* = 65.5 years; *SD* = 13.2	
		Country of participants: Australia	
		Social identities reported: None	

*Note*. Grade of practice recommendation and descriptions are based on the American Society of Plastic Surgeons: Evidence-based clinical practice guidelines as cited in Burns et al. (2011). CBDI = Cognitive Behavioural Driver’s Inventory; DPAB = Dynavision Performance Assessment Battery; MVPT = motor-free visual perceptual test; SDSA = stroke driver’s screening assessment; VRST = visual recognition slide test.

For the second objective, to critically appraise the representativeness of demographic subgroups within stroke and fitness to drive research, each study in Table 3 included the subgroups used in the test investigation. In addition, we completed analyses of (a) the number and proportion of studies from countries across the globe, (b) the weighted mean age of participants, (c) the number and proportion of female and male participants, (d) demographic information reported across all included studies, and (e) weighted mean length of time since stroke.

## Results

**Figure 1:**
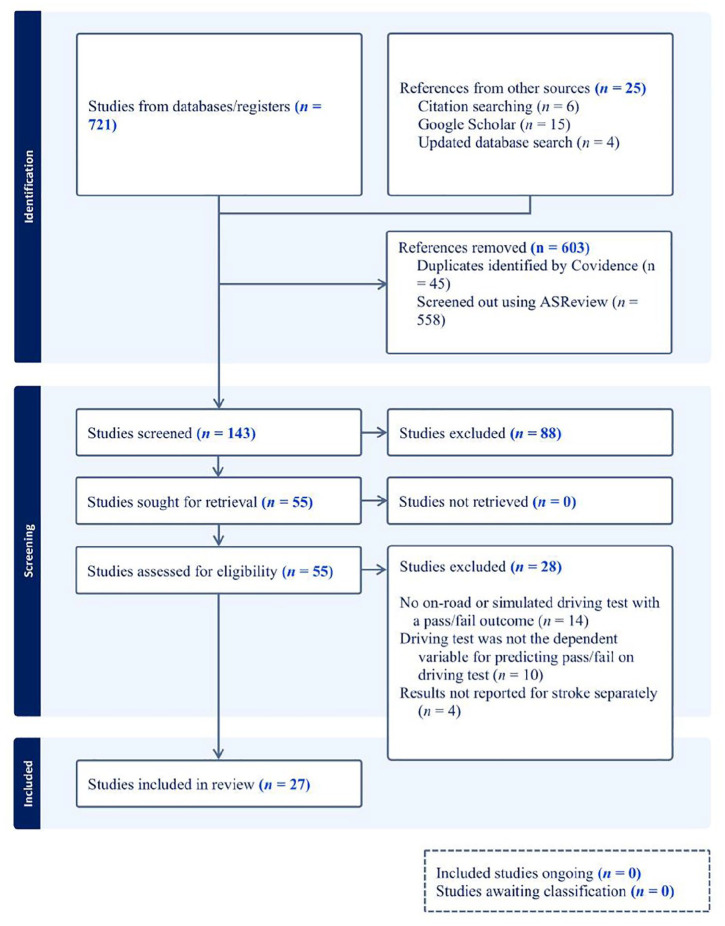
Flow diagram of results of database searches, screening, full-text review, and study inclusion.

Following initial database searches and updated searches, 27 articles were included in the review. For complete details, including reasons for exclusion, see Figure 1 for the PRISMA flow diagram. The most common reasons for exclusion were (a) not including an on-road or simulated driving test with a pass/fail outcome (*n* = 14 papers excluded; examples include Heikkilä et al., 1999; McNamara et al., 2019), or (b) having dependent variables related to driving *performance* rather than a dichotomized pass/fail on a driving test (*n* = 10 papers excluded; examples include Mazer et al., 2003; Stapleton et al., 2012). Summaries of all included studies and quality appraisal ratings are presented in Table 2. The average of the quality appraisal ratings across included studies was 6/9 items, *SD* = 1.1, range 5 to 7. One study not published in English was included in the review (Korean language). We emailed the corresponding author to investigate if any translated copies exist; however, we did not receive a response.

Suggested screening tests and assigned grade of practice recommendation along with descriptions of relevant studies for each study are presented in Table 3. The Stroke Driver’s Screening Assessment (SDSA) and Trail Making Test-B (TMT-B) received the highest grade of practice recommendation in the current review. Overall, studies examining the SDSA (all versions) have tested 410* participants (66 female [16%]) and the weighted mean age of participants was 61.52 years ± 5.9. (*N.B. the total number of participants in studies examining all versions of the SDSA is actually *n* = 520, however, Björkdahl [2015; which included *n* = 110] did not report sex of participants and thus was not included in this calculation). Similarly, studies that found the TMT-B to be predictive of pass/fail driving outcomes included a total of 549 participants (104 female [19%]) and the weighted mean age was 58.2 ± 2.74. Taken together, in the studies examining both measures, men comprised >80% of the sample participants consisting primarily of individuals over the age of 58 years. Moreover, all studies were completed in high-income countries in except for one study conducted in Malaysia (Munin et al., 2023).

A test commonly used in clinical practice which received a lower practice grade recommendation is the Motor-Free Visual Perceptual Test (MVPT). Although the MVPT was previously recommended for driver screening in best practice guidelines (Heart & Stroke Foundation of Canada, 2019), this test was assigned Grade C in the present study. The studies examining the MVPT had inconsistent, low-level evidence for its use, with more studies (3 out of 5) indicating it is not predictive of fitness to drive. Early studies of MVPT pointed to predictive validity, but recent studies have not replicated those findings.

To critically assess the representation of demographic subgroups within the entire body of literature, the demographics of participants in all included studies were analyzed. The results revealed only 21% (*n* = 479/2,270) of the study participants were female, the weighted average age of participants was 61.6 ± 5 years, and 99% (*n* = 2,226/2,246) of the participants live in high-income countries. The mean time since stroke was reported for a very small portion of participants (5%; *n* = 109/2,270), and the weighted mean for time since stroke was 12.3 months (*SD* = 11.6) among reporting studies. For other demographic information, only six (22%; 6/27) included studies reported the education level of participants, two (7%; 2/27) reported language, two (7%; 2/27) reported racial identity and only one (4%; 1/27) reported employment status. Among the six studies that reported specific participant education levels, most participants had post-secondary education (64.5%; *n* = 142), then secondary education (34.5%; *n* = 77) and the fewest with primary education (1%; *n* = 2). However, education level was only described for 10% (*n* = 220/2,270) of participants in included studies. The only participant racial identities reported included Southeast Asian (0.4%; *n* = 9/2,270), South Asian (0.09%; *n* = 2/2,270), Asian (0.4%; *n* = 9/2,270), Caucasian (2.2%; *n* = 50/2,270) and African American (1%; *n* = 22/2270), which indicates the racial identities of 97% (*n* = 2,198/2,270) of participants are not reported. Many studies reported “gender,” but there was no information about gender beyond a male and female binary and thus it is possible that only sex at birth was collected. No studies reported on other variables such as income, or type of employment of participants.

## Discussion

The objectives of this systematic review were to (a) identify screening tests that are predictive of fitness to drive following stroke, and (b) critically appraise the representation of different demographic subgroups in extant fitness to drive research following stroke. The screening tests identified in the present review as most predictive of pass/fail for stroke populations on a driving test are consistent with the findings of previous systematic reviews. Stroke Driver’s Screening Assessment (SDSA; Nouri & Lincoln, 1992) which has American (Akinwuntan et al., 2013), Nordic (Lundberg et al., 2003) and Malaysian versions (Munin et al., 2023) was rated *Grade A* or *Strong Recommendation*. The Trail-Making-Test B (TMT-B; Partington, & Leiter, 1949) was rated *Grade B*, or *Recommendation*. A previous systematic review completed by Marshall et al. (2007) recommended Trail Making Test-B, along with Rey Osterreith Complex Figure (Rey & Osterrieth, 1941) and Useful Field of View (UFoV; Edwards et al., 2006). The systematic review by Hird et al. (2014) also recommended SDSA, UFoV, and Rey Osterreith Complex Figure. Finally, the most extensive review and meta-analysis by Devos et al. (2011) also recommends use of TMT-B, as well as Road Sign recognition test and the Compass Test, which are components of the SDSA.

Although findings regarding SDSA and Trails-B are consistent, other tests such as the Rey Osterreith Complex Figure, Motor-Free Visual Perceptual Test (MVPT; Colarusso & Hammill, 1972), and UFoV were graded as *Level C* or *Option* in this study for their predictive validity of pass/fail driving outcomes. It is likely that these tests were rated at only Level C in the present review due to studies excluded based on the dichotomous pass/fail inclusion criteria. Nonetheless, it appears that tests previously identified as predictive of fitness to drive following stroke have remained stable from the earliest review (Marshall et al., 2007) to present. Importantly, there is a significant caveat to these findings- the tests continue to be investigated using same limited subgroups of stroke populations, namely, the vast majority are older men from high-income countries.

The lack of representation of women, people younger than 55 years of age and people from lower income countries is concerning, considering these are the precise groups in which stroke incidence is on the rise (Ekker et al., 2019; Leppert et al., 2022; Scott et al., 2022). This review provides further evidence of the gender gap in stroke research (Heart & Stroke Association, 2023) and raises important considerations about how evidence supporting these tests is interpreted and communicated. Furthermore, many studies did not report other relevant demographic information of participants including gender, racial identities, level of education, employment status, and income. As these demographics are not reported in the literature, the extent to which many subgroups of people who experience stroke are represented in the literature is unknown. Not only must research include a diversity of participants to accurately reflect the population of people who experience stroke, it is also important to explore if demographics affect driver screening test results to optimize validity, reliability and cultural relevance of tests for specific subgroups (Fujii, 2018).

Stroke best practice guidelines underscore the necessity to use valid and reliable screening tools (Mountain et al., 2020; National Stroke Foundation, 2010; Winstein et al., 2016). Many tests have been validated for stroke as a *diagnostic group*, however much of the existing literature does not report specific participant demographic information. Consequently, diverse subgroup representation of people who experience stroke is not evident in the fitness to drive literature which poses limitations for applying research for diverse patient identities. As with all practice areas, the application of evidence-based practice occurs within the practical confines of existing research, and occupational therapists have expressed concerns about the limitations and applicability of research to clinical practice in general (Upton et al., 2014). Clinical reflection is a useful tool to situate assessment results- when selecting a driver screening tool, therapists are encouraged to reflect on the unique characteristics of the patient and the shortcomings of the literature when interpreting assessment findings (Restall et al., 2022).

The ECLECTIC framework (Fujii, 2018), which emphasizes how characteristics such as income, education, and culture impact neuropsychological testing results, may prove a useful framework within occupational therapy to guide selection of screening tools and interpretation of results. As decisions regarding fitness to drive are highly important to patients and a critical source of contention, optimizing the validity and reliability of assessment strategies is crucial (Betz et al., 2016; Korner-Bitensky et al., 2010; Sangrar et al., 2018). Indeed, a meta-synthesis of patient preferences surrounding driving conversations with health care providers found that patients want providers to include and discuss objective evidence and testing results to substantiate their recommendations (Betz et al., 2016). Thus, adopting frameworks such as ECLECTIC may help to enhance the validity and ultimately acceptance and credibility of health care provider driving recommendations among patients.

Given the limitations in demographic reporting, the current research does not provide sufficient data to wholly apply the ECLECTIC framework for occupational therapy driver screening practices. However, ECLECTIC can be applied to development of *future* primary research to work toward more detailed demographic reporting. Specifically, to implement the ECLECTIC framework in practice (Fujii, 2018), future primary driving research should prioritize (a) detailed reporting of participant demographic information (including income, education, language fluency, racial, and cultural identities) and (b) purposeful recruitment for subgroups of stroke populations not currently represented in research, particularly women, people younger than 55 years of age, people from racialized communities, and people from low-income countries. We also advocate for collecting and reporting of both sex and gender, as it is unclear how each impact driving related behaviors and performance on clinical tests, which warrants further inquiry (Keay et al., 2018; Özkan & Lajunen, 2006). In addition, we recommend reporting participant employment status, type of work, and working hours (e.g., shiftwork), as work-related factors also affect driving-related outcomes (Knott et al., 2020; Lyu et al., 2018). Moreover, both gender and employment factors provide further socio-cultural description of participants to inform decisions about the clinical applicability of research findings (Fujii, 2018; Restall et al., 2022).

Funders and policymakers are encouraged to incentivize such research priorities to address gaps in predicting fitness to drive after stroke. More clarity surrounding demographic details about participants will also increase transparency and awareness of groups included (and excluded) in research studies to inform questions of applicability of results for specific patient subpopulations. Most of the research was conducted with patients later in their stroke recovery trajectories (12.3 months post-stroke; *SD* = 11.6) and as such, more research is also needed with patients <1 year after stroke, when fitness to drive determinations are often made (when leaving acute care or rehabilitation settings; Mountain et al., 2020).

### Limitations

The present review sought to include studies that evaluate fitness to drive using a dichotomized pass/fail criterion on a driving test. Although pass/fail driver tests are the clinical gold standard in driving assessment practice (Bédard & Dickerson, 2014), some research was not included that may still contribute to the evidence predicting driving ability or performance following stroke. Similarly, research with diagnostically heterogeneous samples (or where results for stroke were not reported separately) were excluded from the review (e.g., Krishnasamy & Unsworth, 2011; Unsworth et al., 2012). Stroke best practice guidelines indicate screening tests should be validated for stroke populations (Mountain et al., 2020). As such, other relevant studies/screening tools for driver fitness with other diagnostic groups (such as dementia) were excluded from this review (e.g., Eramudugolla et al., 2022 [evaluated the Mini-Mental State Examination]; Kwok et al., 2015 [evaluated the Montreal Cognitive Assessment]).

Another related limitation of the current review (and systematic reviews in general) is that the application of very specific inclusion criteria can perpetuate the same studies and findings over time (Greenhalgh et al., 2018). Indeed, the current review revealed similar findings to previous reviews and included similar studies. The research question for the current study is specific and thus is coherent with the strict inclusion criteria of a SLR methodology (Greenhalgh et al., 2018); however, a *narrative* review may be a useful future direction to explicate the findings of this work, particularly surrounding subgroup representation.

Finally, artificial intelligence generated translation was utilized to complete some data extraction. The gold standard for using translated works is to procure costly professional translations. However, using creative strategies to include literature may represent a cost-effective way forward to address the English language privilege in systematic reviews (Walpole, 2019).

A limitation in the body of evidence is that most studies did not provide cut points for fit and unfit to drive. Evidence for fit/not fit thresholds are most useful for clinical practice, but unfortunately, this review was not able to provide updated recommendations. The best evidence for cut point data remains within the systematic review by Devos et al. (2011) which also completed meta-analysis to calculate the cut points. The present study did not plan a meta-analysis and did not request access to raw data from included studies to complete further analysis, and thus data was extracted from papers *as reported*. Further research is warranted to examine cut offs for driver fitness. As clinicians are often tasked to make dichotomous decisions, such as if a report is required to be sent to the local driver’s licensing authority or not (Canadian Council of Motor Transport Administrators, 2020), research that mirrors the same dichotomous dependent variable is needed.

## Conclusion

This systematic review found that although many of the recommended screening tests for driver fitness after stroke have remained stable overtime, the subgroups reported to be represented in the literature are consistently older men from high-income countries. Furthermore, research studies rarely provide detailed demographic data to characterize the subgroup representation within study samples, which further obscures the representativeness of the body of literature. The present review highlighted screening tests recommended in the literature along with the specific subgroups the findings were based on, where reported. More primary research is warranted that (a) reports detailed demographic information about participants and (b) includes participants belonging to demographic subgroups underrepresented in current research.

## Supplemental Material

sj-docx-1-otj-10.1177_15394492251344518 – Supplemental material for Screening Fitness to Drive After Stroke Across Demographic Subgroups: A Systematic Review Supplemental material, sj-docx-1-otj-10.1177_15394492251344518 for Screening Fitness to Drive After Stroke Across Demographic Subgroups: A Systematic Review by April Vander Veen, Leaha Johnston, Jeffrey Holmes, Patricia Tucker and Liliana Alvarez in OTJR: Occupational Therapy Journal of Research

sj-docx-2-otj-10.1177_15394492251344518 – Supplemental material for Screening Fitness to Drive After Stroke Across Demographic Subgroups: A Systematic Review Supplemental material, sj-docx-2-otj-10.1177_15394492251344518 for Screening Fitness to Drive After Stroke Across Demographic Subgroups: A Systematic Review by April Vander Veen, Leaha Johnston, Jeffrey Holmes, Patricia Tucker and Liliana Alvarez in OTJR: Occupational Therapy Journal of Research

sj-docx-3-otj-10.1177_15394492251344518 – Supplemental material for Screening Fitness to Drive After Stroke Across Demographic Subgroups: A Systematic Review Supplemental material, sj-docx-3-otj-10.1177_15394492251344518 for Screening Fitness to Drive After Stroke Across Demographic Subgroups: A Systematic Review by April Vander Veen, Leaha Johnston, Jeffrey Holmes, Patricia Tucker and Liliana Alvarez in OTJR: Occupational Therapy Journal of Research

sj-docx-4-otj-10.1177_15394492251344518 – Supplemental material for Screening Fitness to Drive After Stroke Across Demographic Subgroups: A Systematic Review Supplemental material, sj-docx-4-otj-10.1177_15394492251344518 for Screening Fitness to Drive After Stroke Across Demographic Subgroups: A Systematic Review by April Vander Veen, Leaha Johnston, Jeffrey Holmes, Patricia Tucker and Liliana Alvarez in OTJR: Occupational Therapy Journal of Research
