# Supplementary material for: Screening Fitness to Drive After Stroke Across Demographic Subgroups: A Systematic Review
Source: OTJR (Thorofare N J). 2025 Jun 16;46(3):573–90. doi: 10.1177/15394492251344518 (PMC13219784; doi:10.1177/15394492251344518)
Supplement: sj-docx-2-otj-10.1177_15394492251344518 – Supplemental material for Screening Fitness to Drive After Stroke Across Demographic Subgroups: A Systematic Review [file sj-docx-2-otj-10.1177_15394492251344518.docx]

Supplemental Table 2

*Modified Quality Assessment Checklist (Downs & Black, 1998; Duch et al., 2013)*

| Item number Downs & Black (1998) | Item |
| --- | --- |
| 1 | Is the hypothesis/aim/objective of the study clearly described? |
| 2 | Are the main outcomes to be measured clearly described in the introduction or methods section? |
| 3 | Are the characteristics of the patients included in the study clearly described? |
| 6 | Are the main findings of the study clearly described? |
| 7 | Does the study provide estimates of the random variability in the data for the main outcomes? |
| 11 | Were the subjects asked to participate in the study representative of the entire population from which they were recruited? |
| 12 | Were those subjects who were prepared to participate representative of the entire population from which they were recruited? |
| 18 | Were the statistical tests used to assess the main outcomes appropriate? |
| 20 | Were the main outcome measures used accurate (valid and reliable)? |
| 10* | Have actual probability values been reported (e.g. 0.035 rather than <0.05) for the main outcomes except where the probability value is less than 0.001? |

*Note.* * removed item from Duch et al. (2013)
