# Supplementary material for: Screening Fitness to Drive After Stroke Across Demographic Subgroups: A Systematic Review
Source: OTJR (Thorofare N J). 2025 Jun 16;46(3):573–90. doi: 10.1177/15394492251344518 (PMC13219784; doi:10.1177/15394492251344518)
Supplement: sj-docx-3-otj-10.1177_15394492251344518 – Supplemental material for Screening Fitness to Drive After Stroke Across Demographic Subgroups: A Systematic Review [file sj-docx-3-otj-10.1177_15394492251344518.docx]

Supplemental Table 3

*Grade Practice Recommendations**

| Grade | Descriptor | Qualifying Evidence | Implications for Practice |
| --- | --- | --- | --- |
| A | Strong recommendation | Level I evidence or consistent findings  from multiple studies of levels II, III, or IV | Clinicians should follow a strong recommendation unless a  clear and compelling rationale for an alternative approach is  present |
| B | Recommendation | Levels II, III, or IV evidence and  findings are generally consistent | Generally, clinicians should follow a recommendation but should remain alert to new information and sensitive to patient preferences |
| C | Option | Levels II, III, or IV evidence, but  findings are inconsistent | Clinicians should be flexible in their decision-making regarding appropriate practice, although they may set bounds  on alternatives; patient preference should have a substantial influencing role |
| D | Option | Level V evidence: little or no systematic empirical evidence | Clinicians should consider all options in their decision making and be alert to new published evidence that clarifies the  balance of benefit versus harm; patient preference should have a substantial influencing role |

*From the American Society of Plastic Surgeons: Evidence-based clinical practice guidelines as cited in Burns et al. (2011).
