# Supplementary material for: Screening Fitness to Drive After Stroke Across Demographic Subgroups: A Systematic Review
Source: OTJR (Thorofare N J). 2025 Jun 16;46(3):573–90. doi: 10.1177/15394492251344518 (PMC13219784; doi:10.1177/15394492251344518)
Supplement: sj-docx-4-otj-10.1177_15394492251344518 – Supplemental material for Screening Fitness to Drive After Stroke Across Demographic Subgroups: A Systematic Review [file sj-docx-4-otj-10.1177_15394492251344518.docx]

**Table 2 (and 3) References**

Akinwuntan, A. E., Feys, H., de Weerdt, W., Baten, G., Arno, P., & Kiekens, C. (2006).

Prediction of driving after stroke: A prospective study. *Neurorehabilitation and Neural Repair*, *20*(3), 417–423. <https://doi.org/10.1177/1545968306287157>

Akinwuntan, A. E., Gantt, D., Gibson, G., Kimmons, K., Ross, V., Rosen, P. N., & Wachtel, J. (2013). United states version of the stroke driver screening assessment: A pilot study. *Topics in Stroke Rehabilitation*, *20*(1), 87–94. <https://doi.org/10.1310/tsr2001-87>

Barco, P. P., Wallendorf, M., Snellgrove, C. A., Ott, B. R., & Carr, D. B. (2014). Predicting road test performance in drivers with stroke. *The American Journal of Occupational Therapy*, *68*, 221–229.

Björkdahl, A., Nilsson, L., & Jönsson, U. (2015). Which is the best way to assess and follow-up fitness to drive after stroke? *Physical Medicine and Rehabilitation-International*, *2*(6), 1054.

Bouillon, L., Mazer, B. L., & Gélinas, I. (2006). Validity of the Cognitive Behavioural Driver’s Inventory in predicting driving outcome. *American Journal of Occupational Therapy*, *60*(4), 420–427.

Chua, M., McCluskey, A., & Smead, J. M. (2012). Retrospective analysis of factors that affect driving assessment outcomes after stroke. *Australian Occupational Therapy Journal*, *59*(2), 121–130. https://doi.org/10.1111/j.1440-1630.2012.01005.x

George, S., Clark, M., & Crotty, M. (2008). Validation of the Visual Recognition Slide Test with stroke: A component of the New South Wales occupational therapy off-road driver rehabilitation program. *Australian Occupational Therapy Journal*, *55*(3), 172–179. https://doi.org/10.1111/j.1440-1630.2007.00699.x

George, S., & Crotty, M. (2010). Establishing criterion validity of the useful field of view assessment and stroke drivers’ screening assessment: Comparison to the result of on-road assessment. *American Journal of Occupational Therapy*, *64*(1), 114–122. https://doi.org/10.5014/ajot.64.1.114

Holowaychuk, A., Parrott, Y., & Leung, A. W. S. (2020). Exploring the predictive ability of the motor-free visual perception test (MVPT) and trail making test (TMT) for on-road driving performance. *American Journal of Occupational Therapy*, *74*(5). https://doi.org/10.5014/ajot.119.040626

Kim, J. J., Choi, H., Lim, M. H., Lee, J. A., Han, H. S., Bae, J. H., Lee, J. J., Kim, M. H., Kim, H. L., & Kim, D. A. (2018). The reliability and validity of Revised Cognitive Perceptual Assessment for Driving (CPAD2). 재활복지공학회논문지, *12*(3), 212–222. https://www.dbpia.co.kr/Journal/articleDetail?nodeId=NODE07526309

Klavora, P., Gaskovski, P., Martin, K., Forsyth, R. D., Heslegrave, R. J., Young, M., & Quinn, R. P. (1995). The effects of Dynavision rehabilitation on behind-the-wheel driving ability and selected psychomotor abilities of persons after stroke. *The American Journal of Occupational Therapy*, *49*(6), 534–542. <https://doi.org/10.5014/ajot.49.6.534>

Klavora, P., Heslegrave, R. J., & Young, M. (2000). Driving skills in elderly persons with stroke: Comparison of two new assessment options. *Archives of Physical Medicine and Rehabilitation*, *81*(6), 701–705. <https://doi.org/10.1053/apmr.2000.6285>

Kobayashi, Y., Omokute, Y., Mitsuyama, A., Takaoka, Y., Takama, C., & Watanabe, Y. (2017). Predictors of track test performance in drivers with stroke. *Turkish Neurosurgery*, *27*(4), 530–536. https://doi.org/10.5137/1019-5149.JTN.17358-16.1

Korner-Bitensky, N. A., Mazer, B. L., Sofer, S., Gelina, I., Meyer, M. B., Morrison, C., Tritch, L., Roelke, M. A., & White, M. (2000). Visual testing for readiness to drive after stroke: A multicenter study. *American Journal of Physical Medicine and Rehabilitation*, *79*(3), 253–259. https://doi.org/10.1097/00002060-200005000-00007

Lundberg, C., Caneman, G., Samuelsson, S. M., Hakamies-Blomqvist, L., & Almkvist, O. (2003). The assessment of fitness to drive after a stroke: The Nordic Stroke Driver Screening Assessment. *Scandinavian Journal of Psychology*, *44*(1), 23–30. https://doi.org/10.1111/1467-9450.00317

Lundqvist, A., Gerdle, B., & Rönnberg, J. (2000). Neuropsychological aspects of driving after a stroke-in the simulator and on the road. *Applied Cognitive Psychology*, *14*(2), 135–150. https://doi.org/10.1002/(SICI)1099-0720(200003/04)14:2<135::AID-ACP628>3.0.CO;2-S

Mazer, B. L., Korner-Bitensky, N. A., & Sofer, S. (1998). Predicting ability to drive after stroke. *Archives of Physical Medicine and Rehabilitation*, *79*(7), 743–750. https://doi.org/10.1016/S0003-9993(98)90350-1

Munin, N. F. A., Mazlan, M., Theivanthiran, S., Aziz, N. A., Isa, A. M., & Abd Rahman, N. H. (2023). Adaptation of The Stroke Driver Screening Assessment (SDSA) to Malaysian Version (MySDSA) and its validation for fitness to drive after stroke. *ASEAN Journal of Rehabilitation Medicine*, *33*(1). https://he01.tci-thaijo.org/index.php/aseanjrm/article/view/254836

Nouri, F. M., Tinson, D. J., & Lincoln, N. B. (1987). Cognitive ability and driving after stroke. *Disability and Rehabilitation*, *9*(3), 110–115. https://doi.org/10.3109/03790798709166334

Nouri, F. M., & Tinson, D. J. (1988). A comparison of a driving simulator and a road test in the assessment of driving ability after a stroke. *Clinical Rehabilitation*, *2*(2), 99–104. https://doi.org/10.1177/026921558800200202

Nouri, F. M., & Lincoln, N. B. (1992). Validation of a cognitive assessment: Predicting driving performance after stroke. *Clinical Rehabilitation*, *6*(4), 275–281. https://doi.org/10.1177/026921559200600402

Nouri, F. M., & Lincoln, N. B. (1993). Predicting driving performance after stroke. *Medical Journal* (Vol. 307, Issue 6902). https://www.jstor.org/stable/29720787

Selander, H., Johansson, K., Lundberg, C., & Falkmer, T. (2010). The nordic stroke driver screening assessment as predictor for the outcome of an on-road test. *Scandinavian Journal of Occupational Therapy*, *17*(1), 10–17. https://doi.org/10.3109/11038120802714898

Söderström, S. T., Pettersson, R. P., & Leppert, J. (2006). Prediction of driving ability after stroke and the effect of behind-the-wheel training. *Scandinavian Journal of Psychology*, *47*(5), 419–429. https://doi.org/10.1111/j.1467-9450.2006.00550.x

Sommer, M., Heidinger, C., Arendasy, M., Schauer, S., Schmitz-Gielsdorf, J., & Häusler, J. (2010). Cognitive and personality determinants of post-injury driving fitness. *Archives of Clinical Neuropsychology*, *25*(2), 99–117. <https://doi.org/10.1093/arclin/acp109>

Sotokawa, T., Nasu, S., Ikuta, J., & Sonohara, K. (2024). Evaluation of driving fitness using

driving simulators in patients with right-hemisphere damage: An unmatched case-control study. *Topics in Stroke Rehabilitation*, *31*(2), 167–177. https://doi.org/10.1080/10749357.2023.2235797

Unsworth, C. A., Baker, A., Lannin, N., Harries, P., Strahan, J., & Browne, M. (2019). Predicting fitness-to-drive following stroke using the Occupational Therapy–Driver Off Road Assessment Battery. *Disability and Rehabilitation*, *41*(15), 1797–1802. https://doi.org/10.1080/09638288.2018.1445784
